# Lysozyme Resistance in *Streptococcus suis* Is Highly Variable and Multifactorial

**DOI:** 10.1371/journal.pone.0036281

**Published:** 2012-04-30

**Authors:** Paul J. Wichgers Schreur, Christian van Weeghel, Johanna M. J. Rebel, Mari A. Smits, Jos P. M. van Putten, Hilde E. Smith

**Affiliations:** 1 Central Veterinary Institute, Wageningen UR, Lelystad, The Netherlands; 2 Department of Infectious Diseases and Immunology, Utrecht University, Utrecht, The Netherlands; 3 Wageningen Livestock Research, Wageningen UR, Lelystad, The Netherlands; University of Kansas Medical Center, United States of America

## Abstract

**Background:**

*Streptococcus suis* is an important infectious agent for pigs and occasionally for humans. The host innate immune system plays a key role in preventing and eliminating *S. suis* infections. One important constituent of the innate immune system is the protein lysozyme, which is present in a variety of body fluids and immune cells. Lysozyme acts as a peptidoglycan degrading enzyme causing bacterial lysis. Several pathogens have developed mechanisms to evade lysozyme-mediated killing. In the present study we compared the lysozyme sensitivity of various *S. suis* isolates and investigated the molecular basis of lysozyme resistance for this pathogen.

**Results:**

The lysozyme minimal inhibitory concentrations of a wide panel of *S. suis* isolates varied between 0.3 to 10 mg/ml. By inactivating the *oatA* gene in a serotype 2 and a serotype 9 strain, we showed that OatA-mediated peptidoglycan modification partly contributes to lysozyme resistance. Furthermore, inactivation of the *murMN* operon provided evidence that additional peptidoglycan crosslinking is not involved in lysozyme resistance in *S. suis*. Besides a targeted approach, we also used an unbiased approach for identifying factors involved in lysozyme resistance. Based on whole genome comparisons of a lysozyme sensitive strain and selected lysozyme resistant derivatives, we detected several single nucleotide polymorphisms (SNPs) that were correlated with the lysozyme resistance trait. Two SNPs caused defects in protein expression of an autolysin and a capsule sugar transferase. Analysis of specific isogenic mutants, confirmed the involvement of autolysin activity and capsule structures in lysozyme resistance of *S. suis*.

**Conclusions:**

This study shows that lysozyme resistance levels are highly variable among *S. suis* isolates and serotypes. Furthermore, the results show that lysozyme resistance in *S. suis* can involve different mechanisms including OatA-mediated peptidolycan modification, autolysin activity and capsule production.

## Introduction

*Streptococcus suis* is an important pig pathogen causing severe infections including meningitis, septicemia, endocarditis, pneumonia and arthritis. *S. suis* is also a zoonotic agent displaying comparable disease manifestations in humans as are seen in pigs [1], [2], [3]. The host innate immune system is an important factor in the prevention and elimination of *S. suis* infections, the involvement of pattern recognition receptors (PRRs) in sensing *S. suis* has recently been described [4], [5]. However, little is known about the role of effector molecules of the innate immune system in counteracting *S. suis* infections. One important effector molecule, with anti-bacterial activity, is the protein lysozyme.

Lysozyme is found in high concentrations (>500 µg/ml) in several bodily secretions including tears, mucus, milk and saliva [6], [7]. In addition neutrophil granules contain significant amounts of the protein [8], [9], [10]. Lysozyme weakens bacterial peptidoglycan layers by hydrolysis of the 1,4-beta-linkages between *N*-acetylmuramic acid (NAM) and *N*-acetyl-D-glucosamine (NAG) residues. Extensive hydrolysis results in bacterial lysis. Deficiencies in lysozyme, by gene inactivation, have been shown to increase susceptibility to streptococcal disease [11].

To survive in hostile environments comprising high levels of lysozyme, bacteria have evolved mechanisms to resist lysozyme digestion. In streptococcal species the peptidoglycan modifying *N*-acetylglucosamine deacetylase PgdA and the peptidoglycan *O*-acetyltransferase OatA (designated Adr in *S. pneumoniae* [12]), confer lysozyme resistance. Both enzymes directly change the NAM and NAG structures of peptidoglycan and reduce lysozyme affinity to the peptidoglycan layer [12], [13], [14]. In addition, the tRNA dependent ligases MurM and MurN, encoded by the *murMN* operon, can increase resistance to lysozyme by introducing extra peptide cross-linking in the peptidoglycan layer [15], [16]. Occasionally, such molecular changes to the peptidoglycan layer are accompanied with changes in bacterial morphology [17].

For *S. suis* the lysozyme sensitivity between and within serotypes has not been investigated systematically. Furthermore, limited data is available about the involvement of peptidoglycan modifying enzymes of *S. suis* in lysozyme resistance. So far, only the role of a PgdA homologue in lysozyme resistance of a serotype 2 strain has been reported [18]. In the latter study, an isogenic *pgdA* mutant showed an unaltered lysozyme resistance phenotype compared to wild type bacteria *in vitro* and a strongly reduced virulence *in vivo*. The genome sequences of various *S. suis* serotype 2 strains suggests the presence of an *O*-acetyltransferase (OatA) gene and the absence of a pneumococcal *murMN* operon homologue in *S. suis* [19], [20]. However, recent sequence analysis of a *S. suis* serotype 9 strain suggests the existence of a *S. suis murMN* operon [21].

The objective of the present study was to determine lysozyme resistance levels in a panel of *S. suis* isolates and to investigate the molecular basis of this resistance. To accomplish this, we focussed on homologues of well-known peptidoglycan modifying enzymes. In addition, we used an unbiased approach based on comparative whole genome analysis.

## Results

### Heterogeneous lysozyme resistance levels in *S. suis*

**Figure 1:**
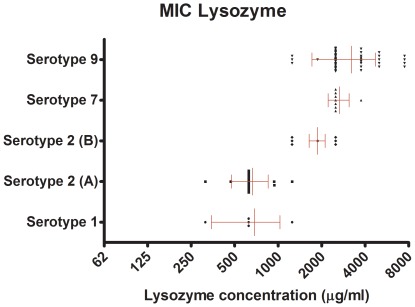
Lysozyme MIC levels of *S. suis*. Wild type serotype 1, 2, 7 and 9 isolates (Table S2) were spotted onto Colombia agar plates containing two-fold increasing concentrations of lysozyme (start concentration: 62.5 µg/ml). Growth was assessed 24 h later and MICs were determined. Serotype 2 strains were separated into two (A and B) different genetic clusters based on CGH data [22]. Each isolate is represented by a dot and one dot represents the mean of two independent observations. The red lines represent the mean resistance level and SD of the indicated groups.

To investigate lysozyme resistance levels in the *S. suis* species, the lysozyme minimal inhibitory concentration (MIC) was determined for a broad panel of *S. suis* isolates belonging to serotypes 1, 2, 7 or 9. The lysozyme MICs varied between 0.3 and 10 mg/ml, as measured by a plate assay (Fig. 1 and Table S1). In general, isolates belonging to serotypes 7 and 9 resisted higher levels of lysozyme compared to serotype 1 isolates and the majority of the serotype 2 isolates. The differences in lysozyme resistance among the serotype 2 isolates correlated with clusters A and B identified by comparative genome hybridization (CGH) for this serotype [22]. Taken together, these results indicate that lysozyme resistance varies between and within different *S. suis* serotypes and suggest that resistance levels correlate with serotype-related genetic backgrounds.

### Distribution of genes that encode peptidoglycan modifying enzymes

To assess the presence of the *oatA* gene and the *murMN* operon in *S. suis*, we performed gene specific PCRs on the same panel of *S. suis* isolates used above. PCRs yielded products of the expected size for the *oatA* gene in all tested isolates (Table 1, Table S1), suggesting that *oatA* is widely distributed in the *S. suis* species. A putative *murMN* operon (presence product of the expected size) could be detected in about half of the isolates tested. The putative *murMN* operon is predominantly present in serotype 9 isolates with relative high lysozyme resistance levels, however the majority of relatively high lysozyme resistant serotype 7 and 2 isolates lack the operon. Furthermore, the *murMN* operon could not be detected in all lysozyme sensitive serotype 1 and 2 isolates (Table 1, Table S1). Based on these data, no apparent correlation could be detected between *oatA* and/or *murMN* presence and the level of lysozyme resistance.

**Table 1:** Distribution of genes encoding peptidoglycan modifying enzymes in different *S. suis* serotypes.

Serotypes	*oatA*	*murMN*
1	(5/5)	(0/5)
2 (CGH A)	(19/19)	(0/19)
2 (CGH B)	(7/7)	(4/7)
7	(8/8)	(1/8)
9	(55/55)	(53/55)

Serotype 2 strains were separated into two (A and B) different genetic clusters based on CGH data [22]. The numbers between brackets represent the number of positive PCR products compared to the total number of analyzed isolates.

### *OatA* but not *MurM* and *MurN* contributes to lysozyme resistance

**Figure 2:**
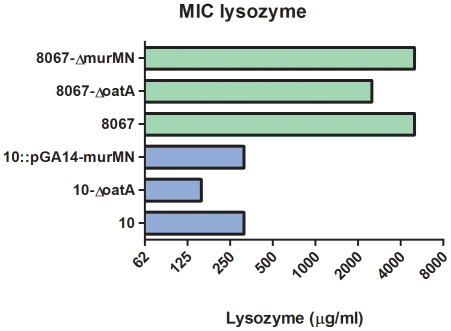
Lysozyme MIC levels of OatA and MurMN mutants. Strains 10-Δ*oatA*, 8067-Δ*oatA*, 8067-Δ*murMN*, 10::pGA14-*murMN* and wild type strain 10 and 8067 were spotted onto Colombia agar plates containing two-fold increasing concentrations of lysozyme. Growth was assessed 24 h later and MICs were determined. Green bars represent wild type and mutant derivatives of serotype 9 strain 8067 and blue bars represent wild type and mutant derivatives of serotype 2 strain 10. Values represent the mean of three independent observations. No error bars are displayed since the MIC values were identical in replicate experiments.

To investigate the role of cell wall modification to *S. suis* lysozyme resistance in more detail, we constructed isogenic *oatA* and *murMN* mutants. *OatA* and *murMN* mutant strains were constructed of serotype 9 strain 8067 (which displays low virulence in pigs) [22], and an *oatA* mutant strain and a *murMN* complemented strain were constructed of serotype 2 strain 10 (which displays high virulence in pigs) [23]. The MIC towards lysozyme for strain 10 and strain 8067 were respectively 0.3 mg/ml and 2,5 mg/ml. Comparisons of the lysozyme MICs of mutant and parent strains indicated that lysozyme MICs of the 8067-Δ*murMN* mutant and the *murMN* complemented strain 10 (10::pGA14-*murMN*) were identical to those of their parent strains (Fig. 2). However, the *oatA* mutants (10-Δ*oatA* and 8067-Δ*oatA*) displayed increased sensitivity to lysozyme compared to their parent strains (Fig. 2). Interestingly, transcription levels of *oatA*, determined by quantitative real time RT-PCR analysis, were highly similar in serotype 1, 2, 7 and 9 isolates (Table S1). Altogether, these results strongly suggest that OatA increases lysozyme resistance in *S. suis*, although OatA seems not solely responsible for a lysozyme resistant phenotype.

### Selection for lysozyme resistance

**Figure 3:**
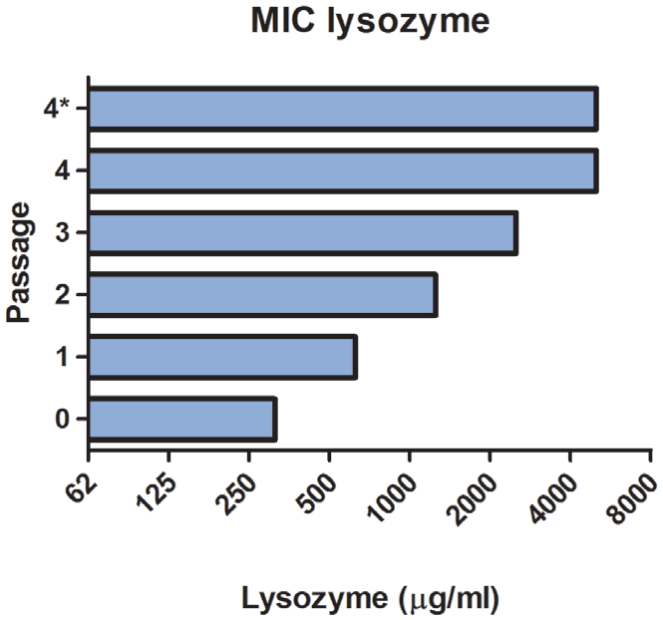
Lysozyme MIC levels of strain 10 after passaging. Lysozyme MIC of strain 10 passaged four times onto Colombia agar plates containing two-fold increasing concentrations of lysozyme (start concentration: 62.5 µg/ml). In general, within 4 passages the lysozyme MIC increased towards levels observed in natural lysozyme resistant strains (compare with Figure 1). * Lysozyme MIC level after sub-culturing lysozyme resistant strains in the absence of lysozyme. Values represent the MIC levels of an example of a selection procedure.

**Figure 4:**
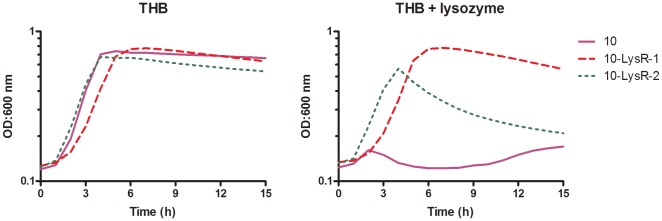
Growth curves of wild type, 10-LysR-1 and 10-LysR-2 *S. suis* strains. Growth of *S. suis* strain 10 (wild type), strain 10-LysR-1 and strain 10-LysR-2 in THB (A) and in THB supplemented with 500 µg/ml lysozyme (B). Values represent the mean of three independent experiments. At almost all time points (three per hour) SD values were maximal 30% of the indicated values.

To discover additional genetic factors involved in lysozyme resistance in *S. suis*, we took advantage of the observation that a lysozyme sensitive strain can acquire higher lysozyme resistance levels by passage in the presence of sub-lethal concentrations of lysozyme. As shown in Fig. 3, the lysozyme sensitive strain 10 (serotype 2) was able to acquire step-wise higher lysozyme resistance levels during passage on plates with successive increasing concentrations of lysozyme. In general, after maximal 4 passages the lysozyme MIC of an isolate was even higher compared to the MIC of the natural lysozyme resistant isolates of serotype 2, 7 and 9 (Fig. 1). In two independent rounds of passaging two lysozyme resistant strains, designated 10-LysR-1, and 10-LysR-2, were obtained (both in three passages). After sub-culturing in the absence of lysozyme, the strains remained equally resistant to lysozyme. For both strains we compared the growth rate in THB with the growth rate in THB supplemented with 500 µg/ml lysozyme. In THB growth rates of strain 10-LysR-1 and 10-LysR-2 roughly resembled those of the wild type strain, although strain 10-LysR-1 displayed an extended lag phase (Fig. 4A). In the presence of lysozyme, only the selected strains 10-LysR-1 and 10-LysR-2 were able to grow efficiently (Fig. 4B), consistent with the selected lysozyme-resistant phenotype.

### Identification of gene polymorphisms associated with increased lysozyme resistance

Since the acquired lysozyme resistance phenotype of strains 10-LysR-1 and 10-LysR-2 remained stable during sub-culture in the absence of lysozyme (Fig. 3), we expected to find changes in the genome sequences of these strains. To identify these genomic alterations we performed Illumina whole genome sequence analysis of strain 10-LysR-1, strain 10-LysR-2 and of parent strain 10. The paired end sequence reads were mapped to reference strain P1/7 [19] and the unmapped reads were assembled *de novo*. Subsequently, single nucleotide polymorphisms (SNPs), insertions and deletions (InDels) of each individual strain were identified relative to reference strain P1/7. Finally, by subtraction, differences in the genome sequences of strain 10-LysR-1, strain 10-LysR-2 and the parent strain 10 were identified. As shown in Table 2, strain 10-LysR-1 and strain 10-LysR-2 both had acquired 3 SNPs during the selection procedure. No insertions or deletions resulting from the selection were detected. The SNPs present in strain 10-LysR-1 were at different loci compared to the SNPs in strain 10-LysR-2, suggesting lysozyme resistance can be acquired via different routes and/or mechanisms. Of the 6 identified SNPs in the two lysozyme resistant strains, 5 were present in protein coding regions and resulted in amino-acid substitutions. In strain 10-LysR-1 one SNP resulted in the amino-acid substitution of His-136-Asn in gene SSU0383 (protein phosphatase), one SNP resulted in the substitution of Arg-215-Ser in gene SSU1292 (membrane protein), and one resulted in the substitution of the start codon (Met-1-Ile) of gene SSU0475 (glycosyl hydrolase family protein; putative autolysin). In strain 10-LysR-2 one SNP resulted in the substitution of Thr-138-Ile in SSU1566 (TrkA family transport protein) and one resulted in the introduction of a stop codon (Leu-211-Stop) in the Cps2E protein (SSU0519, sugar transferase involved in capsule synthesis). The third SNP was located in a non-coding region between gene SSU0319 and gene SSU0320.

**Table 2:** Genomic differences observed between parent strain 10 and its selected lysozyme resistant derivatives 10-LysR1 and 10-LysR-2.

*Strain*	Reference Position	Variation Type	Reference	Allele Variations	Gene in P1/7	Amino Acid Change
10-LysR-1	409567	SNP	C	A	SSU0383	His136Asn
	507081	SNP	C	T	SSU0475	Met1Ile
	1323604	SNP	G	T	SSU1292	Arg215Ser
10-LysR-2	339081	SNP	T	G		
	557107	SNP	T	G	SSU0519	Leu211Stp
	1571782	SNP	C	T	SSU1566	Thr138Ile

### SSU0475 (autolysin) and SSU0519 (capsule) involved in lysozyme resistance

**Figure 5:**
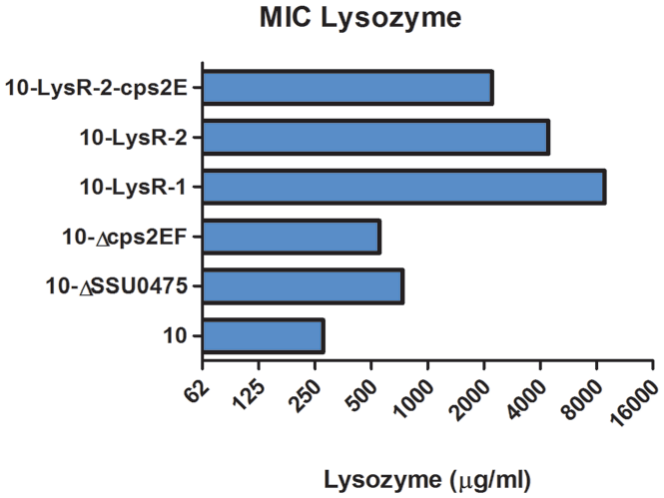
Lysozyme MIC levels of autolysin and capsule mutant strains. Strain 10 (wild type), strain 10-LysR-1, 10-LysR-2, 10-Δ0475, 10-Δ*cps2EF* and 10-LysR-2-*cps2E* were spotted onto Colombia agar plates containing two-fold increasing concentrations of lysozyme. Growth was assessed 24 h later and MICs were determined. Values represent three independent observations. No error bars are displayed since the MIC values were identical in replicate experiments.

From the 6 identified SNPs, the SNPs in SSU0475 (putative autolysin) and SSU0519 (*cps2E*) putatively result in defects in protein expression due to an inactivated start codon and the introduction of a premature stop codon, respectively. To demonstrate the power of this unbiased search for genes involved in a particular trait and to verify the role of these genes in lysozyme resistance, we selected the latter two genes and tested isogenic mutants with defects in gene SSU0475 (10-Δ0475) and gene SSU0519 (capsule mutant, 10-Δ*cps2EF*) [24] for lysozyme resistance. In addition we tested the lysozyme resistant phenotype of strain 10-LysR-2 complemented with an intact copy of the SSU0519 gene. We were unable to introduce an intact copy of SSU0475 into strain 10-LysR-1. As shown in Fig. 5 the lysozyme MICs of strains 10-Δ0475 and 10-Δ*cps2EF* were increased compared to wild type strain 10 and thus resembled the phenotypes of the passage-selected resistant strains. In contrast introduction of an intact copy of gene SSU0519 in strain 10-LysR-2 caused a decrease in lysozyme resistance compared to the selected resistant parent strain, confirming the involvement of the *cps2E* gene in lysozyme resistance. Altogether these results indicate that both SSU0475 and SSU0519 are involved in lysozyme resistance. Since the level of lysozyme resistance of 10-Δ0475 and 10-Δ*cps2EF* was increased but not as high as observed for strains 10-LysR-1 and 10-LysR-2, it is tempting to speculate that the other identified SNP containing genes are involved in lysozyme resistance as well.

### Identified SNPs and genes affect bacterial morphology

**Figure 6:**
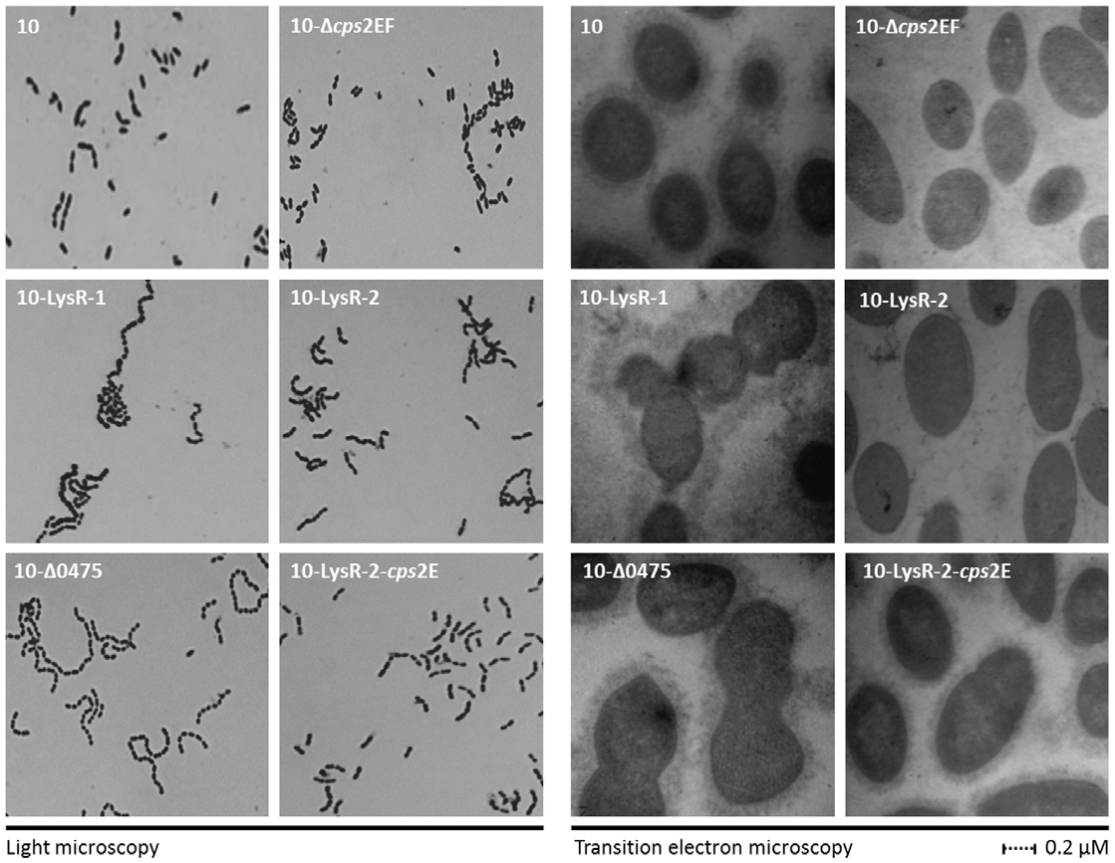
Bacterial morphology lysozyme resistant strains. Strain 10 (wild type), 10-LysR-1, 10-LysR-2, 10-Δ0475, 10-Δ*cps2EF* and 10-LysR-2-*cps2E* were grown exponentially in THB and visualised using crystal violet and light microscopy (A) and TEM (B).

Whether the identified SNPs affect more than solely the observed increase in lysozyme resistance, we tested whether the SNPs caused changes in bacterial morphology. Hereto strain 10, strain 10-LysR-1, strain 10-LysR-2, strain 10-Δ0475, strain 10-Δ*cps2EF* and strain 10-LysR-2-*cps2E* were stained with crystal violet and examined by light microscopy and processed for viewing by transition electron microscopy (TEM). Both light microscopy (Fig. 6A) and TEM (Fig. 6B) of strain 10-LysR-1 revealed increased bacterial chain lengths and cluster formation and more bacterial cell shape diversity compared to the wild type strain. Strain 10-Δ0475 showed also increased chain lengths and heterogeneity in bacterial shape. No major differences in chain lengths were observed between strain 10-LysR-2, strain 10-LysR-2-*cps2E* and the wild type strain. However, TEM analysis clearly indicated reduced amounts of capsule for strain 10-LysR-2 and (as expected) for the isogenic mutant strain 10-Δ*cps2EF*. Thus acquiring lysozyme resistance may be accompanied by alterations (such as loss of classical cell shape or capsule) that may change bacterial behaviour and characteristics besides lysozyme resistance.

## Discussion

In this study we showed lysozyme resistance levels in the *S. suis* species are highly variable. Furthermore with the use of two distinct approaches we identified and characterized factors involved in lysozyme resistance in *S. suis*. The first approach was based on investigating homologues of well-known peptidoglycan modifying enzymes present in other Gram-positive species, and the second (unbiased) approach involved comparative whole genome analysis of a lysozyme sensitive strain and selected lysozyme resistant derivatives. With the use of isogenic mutants we provided convincing evidence that the OatA enzyme of *S. suis* is involved in increasing lysozyme resistance and that autolysin activity and capsule production may also be linked to variation in lysozyme resistance.

Here we provided conclusive evidence based on isogenic *oatA* mutants that OatA, in contrast to PgdA [18], is partly involved in lysozyme resistance in *S. suis* in both relatively lysozyme resistant isolates and in relatively lysozyme sensitive isolates. The OatA protein of *S. suis* serotype 2 strain 10 (relatively lysozyme sensitive) and serotype 9 strain 8067 (relatively lysozyme resistant) used in this study share >95% amino-acid sequence identity with homologous proteins in other *S. suis* isolates present in the NCBI database. Furthermore, *S. suis* OatA proteins share around 50% protein sequence identity with the Adr proteins (OatA homologous) of *S. pneumoniae*. It seems that the OatA protein in the *S. suis* species is relatively conserved, though we cannot exclude that amino-acid differences between different *S. suis* OatA proteins influence protein functionality or activity. The *oatA* gene is also widely distributed in probably all *S. suis* serotypes and expressed at similar levels independent of the *S. suis* background. Overall, we expect that OatA mediated lysozyme resistance is a common and widespread phenomenon in *S. suis*. The contribution of *S. suis* OatA to lysozyme resistance is in agreement with observations in *Staphylococcus aureus* [25], [26], *Listeria monocytogenes* [27], and *S. pneumoniae* [12], [14], emphasizing OatA is an important factor involved in lysozyme resistance in Gram positive species.

In our experiments, no changes in lysozyme resistance due to the presence of the *murMN* operon was observed in *S. suis*, in contrast to observations in *S. pneumoniae* [15], [16]. Possibly, genetic differences in *murMN* sequences of *S. suis* compared to *S. pneumoniae* (45% protein sequence homology) or the bacterial background in which *murMN* is expressed might influence lysozyme resistance phenotypes in *S. suis*.

Using a comparative genome sequence analysis approach, based on specifically selected strains, we were successful with the identification of genetic factors contributing to lysozyme resistance. With this approach we identified and characterized autolytic activity and capsule as important mediators reducing lysozyme resistance in *S. suis*. To our knowledge this kind of approach is the first described to identify factors involved in lysozyme resistance. Besides investigating factors involved in lysozyme resistance, similar approaches may be useful to identify factors and understand mechanisms involved in other phenotypic characteristics like bacteriophage resistance, and antimicrobial peptide resistance.

The effect of increased lysozyme resistance in the presence of reduced autolytic activity strongly suggests *S. suis* autolysins acts in synergy with lysozyme to cause bacterial lysis. Similar to lysozyme, autolysins are able to break down the β(1,4) bond between the NAM and NAG residues, facilitating daughter cell separation. Synergistic effects of autolysin and lysozyme have been reported in *S. pneumoniae* [28]. Interestingly, autolysin activity depends on bacterial growth phase and is tightly regulated by the presence of teichoic acids. Therefore the level and the structure of teichoic acids might influence lysozyme resistance as well, as has been shown in *S. aureus* [29].

The increased resistance to lysozyme of capsule-deficient *S. suis* strains was unexpected. It may be assumed that in the absence of capsule the bacterial peptidoglycan is more easily accessible for lysozyme. One possible explanation for our unexpected finding is that in the absence of capsule there might be increased activity of peptidoglycan modifying enzymes (such as OatA), resulting in increased lysozyme resistant phenotypes. This theory is in agreement with a previous observation in *S. pneumoniae* in which *pgdA* and *adr* mutants displayed thicker capsules [12]. It can be speculated that modifications of the peptidoglycan structure negatively affect the amount of capsule produced and *vice versa*.

Since increased resistance to lysozyme can be acquired by just a few SNPs as evidenced in this study, it is expected that persistence of lysozyme-sensitive isolates in host-environments containing high levels of lysozyme, such as the upper respiratory tract, would be low. On the other hand, the observed differences in bacterial morphology and growth between the lysozyme resistant derivatives: strain 10-LysR-1 and 10-LysR-2, compared to the parent strain suggest acquiring lysozyme resistance reduces overall bacterial fitness. Non-encapsulated *S. suis* mutants are non-virulent [24] and autolysin mutants are attenuated in virulence in other Streptococci [30], [31]. The results are consistent with a scenario that acquiring lysozyme resistance facilitates *S. suis* colonization, but decreases the ability of the pathogen to cause systemic disease. The negative correlation between lysozyme resistance and virulence is in agreement with the observed MIC values of wild type *S. suis* strains. Most lysozyme sensitive isolates belong to the generally highly invasive serotypes 1 and 2 [6], while most of the lysozyme resistant isolates belong to serotype 9, which are in general effective colonizers of the upper respiratory tract [5], [6], [7]. A similar negative correlation between lysozyme resistance and virulence has been suggested for *S. pneumoniae*.

Although our experiments clearly showed involvement of OatA, autolysin and capsule in lysozyme resistance, some other uncharacterised factors are most probably involved in lysozyme resistance in *S. suis* as well. Specific candidates include the additional identified genes in the lysozyme resistance selection procedure (which also contained SNPs), besides the autolysin and the capsule transferase. Especially gene SSU0383, encoding a protein phosphatase, is of increased interest since a homologue of the protein has recently been described to affect bacterial morphology in a serotype 9 isolate [32]. Furthermore, we cannot exclude that additional selection procedures will result in the identification of other unknown factors involved in lysozyme resistance.

**Figure 7:**
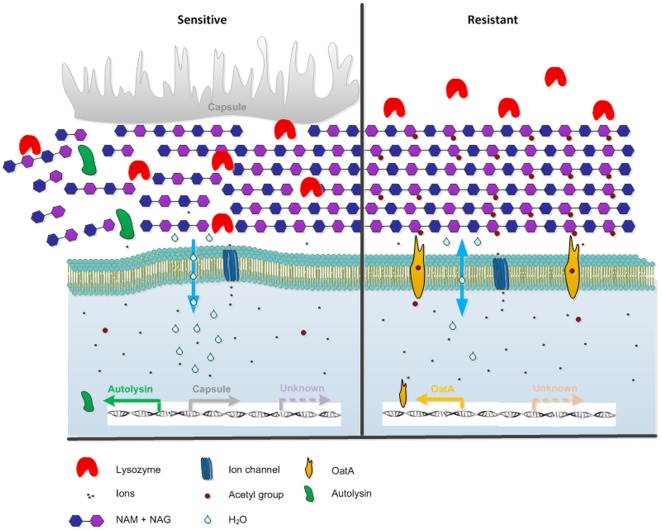
Lysozyme resistance model of *S. suis*. *S. suis* bacteria are expected to resist the antimicrobial activity of lysozyme efficiently in the absence or reduced expression of capsule, in the presence of the peptidoglycan modifying enzyme OatA, and during reduced activity of autolysins. Loss of peptidoglycan stability, due to lysozyme digestion, makes *S. suis* increased vulnerable for osmotic pressure resulting in the flow of water into the bacterium's cytoplasm finally resulting in bacterial lysis.

Overall, this study has gained conclusive evidence that the lysozyme resistant phenotype of *S. suis* involves multiple factors, including those responsible for increased resistance and those responsible for reduced resistance. As displayed in a model (Fig. 7) we hypothesize that, peptidoglycan modification, autolysin activity, and the level of peptidoglycan associated structures such as capsule, are closely related and as a whole determine the level of lysozyme resistance. For example, bacteria with low capsule expression, low activity of autolysins and expressing peptidoglycan modifying enzymes such as OatA are most capable to resist high levels of lysozyme, nevertheless acquiring lysozyme resistance might affect bacterial morphology and overall bacterial fitness and/or virulence.

## Materials and Methods

### Bacterial strains

We used a panel of *S. suis* serotype 1, 2, 7, and 9 isolates as depicted in Table S1. Wild type bacteria, isogenic mutants and complemented mutant strains were grown on Colombia agar plates (Oxoid Ltd, London, United Kingdom) containing 6% horse blood at 5% CO_2_ and 37°C. Bacterial suspensions were grown in Todd-Hewitt broth (THB) (Oxoid Ltd.) for 18 h at 37°C without agitation. *Escherichia coli* were grown on Luria-Bertani (LB) agar plates and in LB broth. Antibiotics were added to culture media at the following concentrations: for *E. coli*, ampicillin 100 µg/ml; chloramphenicol 8 µg/ml and spectinomycin 100 µg/ml; for *S. suis*, chloramphenicol 5 µg/ml and spectinomycin 100 µg/ml when necessary.

### Lysozyme MIC assay

Lysozyme MICs were determined by spotting 8 µl (containing≈5×10^4^ CFU) bacterial suspension in Dulbecco's phosphate buffered saline (D-PBS) on Colombia agar plates containing two-fold increasing concentrations of lysozyme (L6876, Sigma-Aldrich, Zwijndrecht, The Netherlands). Subsequently, growth was evaluated after incubation at 5% CO_2_ and 37°C for 24 h. The minimal concentration in which <5 bacteria were able to grow was designated as the MIC value.

### Detection *oatA*, *murM* and *murN* genes

Overnight cultures in THB were diluted 1∶10 in D-PBS and directly used as template in PCR analysis. Primers (Table S2) used to detect *oatA* (SSU1504 in P1/7), *murM* (SSUD12_0367 in D12) and *murN* (SSUD12_0368 in D12) genes were designed to bind at relatively conserved sequence regions, based on the available *S. suis* genomes in the NCBI database and based on a preliminary *S. suis* serotype 9 genome sequence (H. E. Smith et al., unpublished results). In a final volume of 20 µl, forward and reverse primers (final concentration; 0.25 µM, Table S2) were mixed with 1× Phusion High-Fidelity DNA polymerase master mix (BIOKE, Leiden, The Netherlands) and 2 µl template. PCR conditions were as follows: denaturation for 2 min at 98°C, followed by 35 cycles of 15 s of denaturation at 98°C, 15 s of annealing at 55°C, and 30 s elongation. Amplification of the specific genes was verified on ethidium bromide based 1% agarose gels.

### THB growth

Overnight THB cultures (containing similar amounts of CFUs) of wild type and mutant strains were diluted 1∶100 in 400-µl fresh THB with or without the addition of 500 µg/ml lysozyme. Subsequently, the optical density at 600 nm was followed in time using a Bioscreen C instrument (Thermo Scientific, Breda, The Netherlands) at 37°C.

### Generation 10-Lys-R1 and 10-Lys-R2 strains

Serotype 2 strain 10 (lysozyme sensitive) was plated on Colombia agar plates containing two-fold increasing concentrations of lysozyme and allowed to grow for 24 h at 37°C and 5% CO_2_ (passage one). Subsequently, colonies growing on plates containing the highest lysozyme concentration were collected, re-suspended in 100 µl D-PBS and used to inoculate a new set of plates (passage two). Yet again, colonies growing on plates containing the highest lysozyme concentration were collected and used to inoculate a new set of plates (passage three). After four passages highly lysozyme resistant clones were obtained.

### Construction mutants and complemented mutants

#### General DNA techniques

Chromosomal *S. suis* DNA was isolated as previously described [33]. Phusion High-Fidelity DNA polymerase master mix was used to amplify specific fragments. Plasmid DNA was isolated with the Plasmid DNA Purification System (Promega, Leiden, The Netherlands). DNA purifications were performed with the Zymogen clean up kits (BaseClear, Leiden, The Netherlands). Ligations were performed with T4 DNA ligase (Promega) and ligation mixtures were used to transform *E. coli*. Plasmids were introduced into *S. suis* via electroporation [34]. Primers used in this study are listed in Table S2.

#### Generation of *oatA* and *murMN* mutants

To inactivate the *oatA* gene in serotype 2 strain 10 and serotype 9 strain 8067 and to inactivate the *murMN* operon in strain 8067 we used an inverse PCR strategy. Briefly, primer pairs 1/4 respectively 9/12 were used to amplify chromosomal fragments of the *oatA* and the *murMN* operon with flanking regions of about 0.7–1.5 kb. The fragments were subsequently cloned into pJET1.2 (Fermentas, St. Leon-Rot, Germany) according the manufactures instructions. Subsequently, the generated pJET1.2 plasmids were used as template for an inverse PCR using primer pairs 2/3 or 10/11 to replace an internal fragment by a fragment encoding a spectinomycin resistance mechanism (*spc*, amplified with primers 17/18) as described previously [35], [36]. The *spc* gene was oriented in the same direction as the gene of interest. The resistance cassette containing the flanking regions of *oatA* and/or *murMN* were subsequently amplified using primer pairs 1/4 and 9/12. Finally, these fragments were ligated to the thermo sensitive shuttle vector pSET5 [37], which was linerialized with the *Sma*I restriction enzyme. The pSET5 plasmids were than used to inactivate the *oatA* gene of strain 10 and strain 8067 and the *murMN* operon of strain 8067 as previously described [35], [36] generating 10-Δ*oatA*, 8067-Δ*oatA* and 8067-Δ*murMN*. Mutants were confirmed to have the expected genotype by PCR using primer pairs 5/6, 7/8, 13/14, 15/16. No *oatA* complemented mutants were generated since we did not expected polar effects of *oatA* inactivation because the *oatA* gene is the last gene of the operon and directly downstream the operon, genes are transcribed in the opposite direction from the complementary DNA strand.

#### *MurMN* complementation

To complement strain 10 with the *murMN* operon of strain 8067 we constructed an expression plasmid containing the 8067 *murMN* operon including its putative promoter region. Primers 33 and 34 were used to amplify the *murMN* fragment which was cloned into pJET1.2 generating pJET1.2-*murMN*. Subsequently, the pJET1.2-*murMN* plasmid was digested with *Sal*I and a chloramphenicol resistance gene (*cat*) of pSET5, amplified with primers 15 and 16 and also digested with *Sal*I was cloned upstream. The entire fragment (*murMN*-*cat*) was amplified using primers 35 and 36 and subsequently cloned into pGA14 [38], which is able to replicate in *S. suis*. To do this pGA14 was digested with *HinD*III and *Sac*I, made blunt and ligated to the *murMN*-*cat* PCR fragment, generating pGA14-*murMN*-*cat*. The plasmid was introduced into *S. suis* strain 10 generating 10::pGA14-*murMN*. RNA expression of the *murMN* operon was confirmed by quantitative real time PCR.

#### SSU0475 inactivation

To inactivate SSU0475 (autolysin) in *S. suis* strain 10 we used an overlap extension PCR strategy. Briefly, three fragments (flanking left, *spc*, flanking right) were generated using primers pairs 19/20, 21/22 and 23/24. Subsequently, the three fragments were mixed and used as template in a fusion PCR mixture containing primers 19 and 24. The resulting fragment flanking left-*spc*-flanking right was cloned into pJET1.2 according the manufactures instructions. Subsequently, the plasmid was directly used for electroporation into strain 10 followed by spectinomycin selection. Double cross over mutants were identified by PCR using primer pairs 25/26, 27/28 and 31/32.

#### SSU0519 complementation

To complement strain 10-lysR-2 with an intact copy of SSU0519 (*cps2E*) we constructed a plasmid containing the promoter region and ribosomal binding site of SSU0514 fused with the start codon of SSU0519. In addition the spectinomycin resistance gene (*spc*) was added upstream the fragment for positive selection. Briefly, the three fragments (promoter region SSU0514, SSU0519, *spc*) amplified with primer pairs 37/38, 39/40 and 41/42 were generated and used as template in an overlap extension PCR reaction containing primers 37 and 42. The resulting fragment was subsequently ligated into the thermo sensitive shuttle vector pSET5 [37], which was linerialized with the *Sma*I restriction enzyme. The pSET5 plasmid was subsequently introduced into strain 10-lysR-2 at 37°C allowing single cross over events generating 10-lysR-2-*cps2E*.

### Sequence analysis

Highly purified DNA of parent strain 10, strain 10-lysR-1 and strain 10-lysR-2 was isolated [33] and used for paired end Illumina sequence analysis (BaseClear, Leiden, The Netherlands). The reads were 50 or 75 bp in length and across all bases Illunina quality scores (1.5 encoding) were above 30 and no over-representative k-mers were observed. Of each individual strain the average insert size was about 250 bp. Using CLCbio software, the reads were subsequently mapped against the serotype 2 reference strain P1/7 [19], resulting in a general coverage >100, and unmapped reads were assembled *de novo*. Subsequently, single nucleotide polymorphisms (SNPs), insertions and deletions (InDels) of the individual strains were identified and the genetic changes in 10-LysR-1 and 10-LysR-2, obtained due to the lysozyme resistance selection procedure were identified.

### Morphological analysis

#### Crystal violet staining

Exponentially growing wild type or mutant bacteria (in THB) were heat fixed onto glass slides, washed with water, stained with crystal violet and again washed with water. Bacteria were directly visualized using a Zeiss microscope (1000×).

#### TEM analysis

TEM analysis was performed as previously described with some modifications [39]. Briefly, exponentially growing wild type or mutant bacteria (in 10 ml THB) were harvested by centrifugation, washed with D-PBS and re-suspended in 1 ml D-PBS. Subsequently, bacteria were fixed in cacodylate buffer (0.1 M cacodylate, 5% w/v glutaraldehyde, 0.15% ruthenium red) for 2 h at room temperature, immobilized and pelleted in 2% agarose. One mm^3^ pieces of the bacterial pellet were washed with cacodylate buffer (0.1 M cacodylate) and post-fixed with 2% v/v osmium tetraoxide (overnight at room temperature). Finally, the pieces were dehydrated in graded series of ethanol (50, 70, 95, and 100%) and embedded in Spurr low-viscosity resin (Aurion, Wageningen, The Netherlands) according the manufactures instructions. The samples were cut using a diamond knife and post-stained with uranyl acetate and lead citrate and viewed in an electron microscope (Philips CM 10) at 60 kV.

## Supporting Information

Table S1 Characteristics wild type *S. suis* strains (lysozyme MICs; presence *oatA*, *murMN* and transcription level *oatA*).
(DOCX)

Table S2 Primer sequences.
(DOCX)
