# Supplementary material for: Lysozyme Resistance in Streptococcus suis Is Highly Variable and Multifactorial
Source: PLoS One. 2012 Apr 30;7(4):e36281. doi: 10.1371/journal.pone.0036281 (PMC3340348; doi:10.1371/journal.pone.0036281)
Supplement: Table S1 — Characteristics wild type S. suis strains (lysozyme MICs; presence oatA, murMN and transcription level oatA). (DOCX) [file pone.0036281.s001.docx]

**Table S1. Characteristics wild type *S. suis* strains (lysozyme MICs; presence *oatA*, *murMN* and transcription level *oatA)***

\

CGH cluster: strains were separated into two (A and B) different genetic clusters based on CGH data. ND: not determined. +: fragment of expected size amplified in PCR, -: no fragment of expected size amplified in PCR.
