# Supplementary material for: Lysozyme Resistance in Streptococcus suis Is Highly Variable and Multifactorial
Source: PLoS One. 2012 Apr 30;7(4):e36281. doi: 10.1371/journal.pone.0036281 (PMC3340348; doi:10.1371/journal.pone.0036281)
Supplement: Table S2 — Primer sequences. (DOCX) [file pone.0036281.s002.docx]

**Table S2. Primer sequences**
